# Inhalation of Spray of Tr. Ferri Chloridi in Pulmonary Hemorrhage

**Published:** 1868-12

**Authors:** 


### Inhalation of Spray of Tr. Ferri Chloridi in Pulmonary Hemorrhage.

Surgeon George M. Sternberg, U. S. A., Fort Riley, Kansas, reports, in the *N. Y. Medical Record*, February 1st, 1868, the following case of traumatic pulmonary hæmorrhage, which presents some points of interest, and illustrates one of the uses of Richardson's spray apparatus.

Mr. W., a merchant, aged twenty-eight, while attempting to put a drunken man out of his store, received a stab in the neck, inflicted by a long, narrow-bladed butcher's knife. The knife entered the neck on the right side two and one-half inches above the clavicle, and just to the right of the common carotid artery, cutting the anterior jugular vein, and passing downward and backward into the lung.

The bleeding from the vein was free, but a bystander controlled it by pressure with his thumb, and it did not recur.

Mr. W. at once commenced coughing and expectorating bright red blood, filled with minute bubbles of air. From this time until I was called (about eighteen hours) he continued to cough up every few minutes a mouthful of blood.

When he was first wounded he sent for a hospital steward of the army, with whom he was acquainted. The steward probed the wound, and stated that the œsophagus was wounded. I presume his diagnosis of the case was founded upon the facts that blood came from the mouth, and that any attempt to swallow the medicine he gave produced a violent paroxysm of coughing. The steward prescribed tinct. ergot with a view of controlling the hæmorrhage, and injections of beef-tea and brandy to keep up the pulse. As the hæmorrhage continued and the man was rapidly becoming exhausted, his friends became alarmed and sent for me. After seeing the case, I at once sent for my Richardson's spray producer and some tinct. ferri chlor. The distance was six miles, and it was two hours before my messenger returned. In the meantime Mr. W. continued to cough up a mouthful of blood every few minutes, and the total amount during the two hours could not have been less than sixteen fluid ounces. Perfect rest was enjoined, and no treatment adopted until the spray apparatus arrived. I then added half ounce tinct. ferri chlor. to five ounces of water, and placing the extremity of the instrument well back in the mouth, caused the spray to enter the lungs, by pressing the bulb at each inspiration. This was continued for about a minute, and after an interval of five minutes was again resumed for a minute.

The hæmorrhage was arrested completely, and did not recur. About twenty minutes after a hypodermic injection of morph. sulph. gr. one-fourth was administered, and in a very short time the patient fell into a quiet sleep. I remained with him all night. He occasionally woke up for a moment and dropped asleep again. The next morning he commenced to cough up occasionally a little dark clotted blood, evidently a part of the clot formed by the action of the tinct. ferri chlor.

Perfect rest was enjoined, and injections of beef-tea and brandy were administered from time to time during the next twenty-four hours. The following day he was able to swallow a little wine and beef-tea, and in addition to the small clots of dark blood, a little muco-pus was expectorated.

He continued rapidly to improve, the external wound healing by first intention, and there being no inflammation of the lung beyond the immediate vicinity of the wound. He is now (nine days after the injury) able to sit up in his bed, and to attend to some business.

He lost his voice entirely from the moment he was wounded, and has not yet regained it. He can only swallow liquids, in small quantities at a time, and then by an effort and using great care, as there is a disposition for them to pass into the larynx, producing violent paroxysms of coughing.

These phenomena are probably due to section of the recurrent laryngeal nerve, or some of its branches.—*Half-Yearly Compendium.*

